# Socio-demographic differences in polypharmacy and potentially inappropriate drug use among older people with different care needs and in care settings in Stockholm, Sweden

**DOI:** 10.1177/14034948211018384

**Published:** 2021-06-30

**Authors:** Megan Doheny, Pär Schön, Nicola Orsini, Johan Fastbom, Bo Burström, Janne Agerholm

**Affiliations:** 1 Department of Global Public Health, Karolinska Institutet, Sweden; 2 Aging Research Center, Karolinska Institutet – Stockholm University, Sweden; 3 Center for Epidemiology and Community Medicine, Stockholm County Council, Sweden

**Keywords:** Polypharmacy, potentially inappropriate medication, socio-demographic differences, care needs, integrated care, home-help users, ageing population, dementia, multi-morbidity

## Abstract

**Aims:**

Polypharmacy and potentially inappropriate medications (PIM) are risk factors for negative health outcomes among older people. This study aimed to investigate socio-demographic differences in polypharmacy and PIM use among older people with different care needs in a standard versus an integrated care setting.

**Methods:**

Population-based register data on residents aged ⩾65 years in Stockholm County based on socio-demographic background and social care use in 2014 was linked to prescription drug use in 2015. A logistic regression analysis was used to estimate socio-demographic differences in polypharmacy and PIM, adjusting for education, age group, sex, country of birth, living alone, morbidity and dementia by care setting based on area and by care need (i.e. independent, home help or institutionalised).

**Results:**

The prevalence of polypharmacy and PIM was greater among home-help users (60.4% and 11.5% respectively) and institutional residents (74.4% and 11.9%, respectively). However, there were greater socio-demographic differences among the independent, with those with lower education, older age and females having higher odds of polypharmacy and PIM. Morbidity was a driver of polypharmacy (odds ratio (OR)=1.19, confidence interval (CI) 1.16–1.22) among home-help users. Dementia diagnosis was associated with reduced odds of polypharmacy and PIM among those in institutions (OR=0.78, CI 0.71–0.87 and OR 0.52, CI 0.45–0.59, respectively) and of PIM among home-help users (OR=0.53, 95% CI 0.42–0.67).

**Conclusions:**

Polypharmacy and PIM were associated with care needs, most prevalent among home-help users and institutional residents, but socio-demographic differences were most prominent among those living independently, suggesting that municipal care might reduce differences between socio-demographic groups. Care setting had little effect on inappropriate drug use, indicating that national guidelines are followed.

## Introduction

Inappropriate prescription drug use is a concern for the medication safety among older people, and the rate of such drug use is one indicator of care quality [1]. Frequently, older people experience more complex health problems characterised by multi-morbidity (2+ chronic conditions), age-related changes in pharmacokinetics and pharmacodynamics, as well as functional and cognitive changes that occur with age [1,2]. Pharmaceutical treatment for older people is increasingly complicated by multi-morbidity, which is associated with reduced quality of life, mortality and polypharmacy [2,3].

Polypharmacy is the concurrent use of many drugs, frequently defined by the cut-off of five or more drugs [4]. Prescriptions drugs can be considered potentially inappropriate when the potential harm of a medication outweighs the benefit to the patient [2,5,6]. Generally, the prescribing of medication to older people is largely based on single disease-specific protocols that often neglect other chronic conditions and concurrent prescriptions [5]. Older people exposed to polypharmacy have an increased risk of drug–drug interactions and adverse drug reactions, including falls, reduced quality of life, frailty, avoidable hospitalisations and higher mortality [6–10]. Further, polypharmacy increases the risk of being exposed to potentially inappropriate medications (PIM) [6,11].

Polypharmacy is following an upward trend in Sweden [12] and elsewhere [13,14]. A Swedish study reported that 45% of those aged ⩾75 years experienced polypharmacy, and that the prevalence differed between community-dwelling residents (42%) and those in institutions (69%) [15]. The same pattern was found in another Swedish study for the prevalence of PIM [16]. However, community-dwelling older people are a highly heterogenous group, with different health and care needs, and there are limited studies looking at prescription drug use and comparing those with different care needs, such as those receiving home help compared to those living independently in the community. A Swedish study investigated the impact of medication reviews administered by primary care nurses in Skåne County, included those receiving home help, and reported that the majority of this group had experienced a drug-related problem (DRP), in which 12% was attributed to a PIM [17].

In Sweden, health care and eldercare is universally provided based on the principle of ‘equal access for equal need’ regardless of sex, age and socio-economic position (SEP) [18,19]. Further, prescription drugs are subsidised, and there is a maximum of 2250 SEK out-of-pocket expense per individual in a 12-month period, after which prescriptions are free [20]. However, social inequalities in health-care utilisation still exist [21], and are experienced by older people in need of care with low SEP [22]. Previous studies have indicated that older people with low SEP are prescribed more drugs and to a greater extent exposed to excessive polypharmacy [23–26]. The socio-demographic factors consistently associated with polypharmacy are female sex and lower SEP [24,25], as well as other factors such as multi-morbidityand PIM use [26]. Further, the rate of polypharmacy is high among certain socio-demographic groups such as those with lower education and born outside of Sweden [12].

Swedish eldercare – following an ‘ageing in place’ policy – is financed, organised and provided by the 290 municipalities [27]. Older people must apply for home help from the municipality, and home help is provided based on assessed need, in which the eligibility requirements are determined by a need assessor. There are two main forms of eldercare: home-help services and institutional care. Home help provides two types of support: assistance with domestic household tasks (e.g. shopping, cleaning, laundry, and meals-on-wheels/cooking) and help with personal care (e.g. personal hygiene, dressing and eating). Home help can be complemented by basic home health care (i.e. nurse level) [27]. In Sweden, 72% of older people with long-term care needs received care in their own homes compared to 28% in institutional care [28]. Previous studies on inappropriate drug use have focused on people in institutional care [29–33]. However, there is a limited body of research concerning pharmaceutical drugs use in older people with large care needs who receive home help and those not receiving care.

Integration and coordination between hospitals, primary health care and social care is essential to improve person-centred outcomes and address the complex needs of the older population [34]. As the number of older people living in the community experiencing polypharmacy increases, integrated care (IC) could play a role in the management of prescription drug use [35]. Drug treatment is not ‘one size fits all’ and requires greater coordination, communication and information sharing between doctors, pharmacists, nurses and social care workers, underpinned by informed patient interaction [34,35]. There are many definitions of IC, but we use the following: ‘a coherent set of methods and models on the funding, administrative, organisational, service delivery and clinical levels designed to create connectivity, alignment and collaboration within and between the cure and care sectors’ [36]. In Sweden, the Norrtälje model is an example of an IC system. It has previously been described in detail [37,38].

This study aimed to investigate socio-demographic differences in polypharmacy and PIM use among older people with different care needs (i.e. independent, receiving home help and residents in institutions) in a standard care (SC) compared to an IC setting.

## Methods

We conducted a cross-sectional population-based study using data from 2014 and 2015. The study population consisted of all registered inhabitants aged ⩾65 years living in Stockholm County on 31 December 2014 (*N*=358,528), excluding those with missing education level (*n*=10,189). Those who died during 2015 were included (*n*=13,665). Care needs were measured using the Swedish Social Services Register. For this study, we restricted care need to those receiving home help in the form of personal care in the last six months of 2014 (*n*=10,124) and people registered as living in an institution (*n*=14,445) in 2014. Further, those living in the community and not receiving any form of home help were defined as independent (*n*=291,800).

### Data sources

We obtained socio-demographic variables from the Longitudinal Integration Database for Social Insurance and Labour Market Studies (LISA). This database contains a collection of variables from population registers linked individually through encrypted personal identity numbers. Sex was categorised into male or female. Age was calculated from the registered year of birth and categorised into 65–74, 75–84 or ⩾85 years. Education level was categorised according to the Swedish educational system based on years of education: primary (<9 years), secondary (9–12 years) or post secondary (>12 years). Country of birth was dichotomised into Sweden or other. Living situation was measured using civil status and family type and dichotomised into cohabitating or living alone for people not living in an institution.

The Region Stockholm administrative database (VAL) contains information on all registered out- and inpatient care visits financed by Region Stockholm. We used inpatient care diagnoses to calculate the Charlson Comorbidity Index (CCI) score, which was used to adjust for co-morbididity [42].The CCI assigns scores ranging from 1 to 6 to different morbidities and was calculated for each individual in the study [39]. Dementia diagnosis status was obtained from outpatient data using ICD codes (Appendix A). Care setting was based on area of residence in 2014. People in Norrtälje municipality were defined as living in an IC setting, while those living in the rest of Stockholm County were defined as living in a SC setting.

### Outcomes

The Swedish Prescribed Drug Register (SPDR) holds detailed information on all drugs prescribed and purchased in Sweden. Polypharmacy and PIM were measured by the drugs prescribed during January–March 2015. For people who died during this interval (*n*=3917), we used data on prescribed drugs from October to December 2014. Polypharmacy was defined as being dispensed five or more prescription drugs during a three-month interval. PIM was determined based on an indicator of ‘Drugs that should be avoided unless specific reasons exist’ recommended by the Swedish National Board of Health and Welfare (NBHW) for prescribing to people aged ⩾75 years [40], including long-acting benzodiazepines, medicines with anticholinergic effects, tramadol and propiomazine (Appendix B). PIM was defined as exposure to at least one drug of the four types.

### Ethical considerations

All data were pseudonymised, and the encryption key connecting research identification number to the personal identification number is available only to the authorities responsible for the register data. Ethical permissions were obtained from the Regional Ethics Review Board of Stockholm (Dnr 2016/299–31).

### Analysis

The study population was described by socio-demographics, health and prescription drug use measured in percentages, means and standard deviations. Univarible analysis was perfomed to assess the association between socio-demographic variables (age, sex, level of education, country of birth, living situation, morbidity and care setting) and the risk of polypharmacy as well as being prescribed a PIM stratified by care need group. Multivariable models were used to assess the effect of other variables on polypharmacy and PIM. Variables included in the models were based on the univariable analysis and comparing the Akaike information criterion (AIC) of models. Model 1 adjusted for education, sex and age. Model 2 adjusting for education, sex, age, living alone and born outside of Sweden. Model ) including model 2 and care setting (results are not shown because the odds ratios (ORs) were quite similar to models included in Tables II and III). The comparison of the AIC can be found in Appendix C. Multivariable models presented in Tables II and III were adjusted for level of education, sex, age, living alone, born outside of Sweden, care setting and morbidity. Additional models comparing the different care settings were performed on both outcomes, adjusting for education, sex, age, living situation, country of birth, CCI score, dementia diagnosis and care need group, with the independent group as the reference group. The results are presented as OR and confidence intervals (CI) and were estimated using logistic regression models. The Hosmer–Lemeshow goodness-of-fit statistic was checked to test the fit of the model to the data.

## Results

Home-help users were on average older (83.1 years), mostly female, with primary or secondary education level. More than three-quarters (78.8%) lived alone, and 12.4% had a dementia diagnosis and a higher average CCI score compared to those independent in the community. Residents in institutions were demographically like home-help users, though slightly older (85.4 years), mostly born in Sweden, with primary education and with a similar level of morbidity to home-help users. Further, 31.4% residents in institutions had a dementia diagnosis. In SC, 3.1% of inhabitants received home help compared to 2.8% in IC. However, in IC, there were more residents in institutions (4.7%) compared to SC (4.3%). There were differences in the demographic composition of the inhabitants in the different care settings due to area differences within Region Stockholm (Table I).

**Table I.** Description of those aged ⩾65 years in Stockholm Region and those with different care needs.

	Independent			Home-help users			Residents in institutions		
	All	Care setting		All	Care setting		All	Care setting	
		Standard	Integrated		Standard	Integrated		Standard	Integrated
Variables	(*N*=291,800)	(*N*=278,460)	(*N*=13,340)	(*N*=10,124)	(*N*=9708)	(*N*=416)	(*N*=14,445)	(*N*=13,752)	(*N*=693)
Age (years), *M*±*SD*	73.1±6.6	73.1±6.6	73.4±6.6	83.1±7.8	83.4±8.0	83.2±8.1	85.4±8.0	85.4±8.0	85.0±7.9
Age groups (years)									
65–74	65.1	65.2	63.2	16.6	16.5	18.8	11.9	11.9	12.3
75–84	27.6	27.5	28.9	35.4	35.6	31.2	28.6	28.6	28.6
⩾85	7.3	7.3	7.9	48.0	47.9	50.0	59.5	59.6	59.2
Female	53.4	53.6	50.7	67.9	68.0	64.9	69.8	69.9	67.2
Education level									
Primary	24.2	23.7	34.9	39.0	38.4	51.9	41.9	41.1	57.7
Secondary	43.6	43.5	45.8	39.9	40.2	34.1	40.9	41.3	34.6
Post secondary	32.1	32.8	19.3	21.1	21.4	13.9	17.2	17.7	7.6
Living alone	45.9	46.1	41.7	78.8	78.8	77.6			
Born outside of Sweden	18.9	19.4	10.4	18.7	19.1	8.9	15.8	16.1	10.4
Morbidity									
CCI score, *M*±*SD*	1.13±1.77	1.13±1.76	1.10±1.70	2.23±2.15	2.22±2.15	2.24±2.15	1.88±1.84	1.88±1.84	1.88±1.89
CCI score groups									
0	31.7	31.6	33.2	21.9	22.0	19.0	20.6	20.5	23.2
1–2	18.3	18.2	20.3	38.8	38.6	43.2	45.2	45.2	43.3
⩾3	50.0	50.2	46.5	39.3	39.4	37.7	34.2	34.3	33.5
Dementia diagnosis	1.1	1.1	1.1	12.4	12.4	11.8	31.2	31.3	29.9
Prescription drug use									
Prescriptions, *M*±*SD*	3.0±3.1	3.0±3.1	3.2±3.1	6.0±4.1	5.9±4.1	6.5±3.9	7.2±4.0	7.3±4.1	7.1±2.9
Polypharmacy	25.6	25.5	24.4	60.6	60.4	66.8	74.4	74.4	74.6
PIM	5.2	5.2	5.3	11.5	11.5	13.0	11.9	11.9	11.4

Data shown are percentages unless otherwise indicated.

*SD*: standard deviation; PIM: potentially inappropriate medication; CCI: Charlson Comorbidity Index.

Prescription drug use increased by care need. Those living independently had on average 3.0 drugs, home-help users had 6.0 drugs and residents in institutions had 7.2 drugs (Table I). Accordingly, the prevalence of polypharmacy increased by care need from 25.6% among those who were independent to 60.2% among home-help users and 74.4% among residents in institutions. PIM was more prevalent among home-help users (11.5%) and residents in institutions (11.9%) compared to those living independently (5.2%). Further, there were small differences in the prevalence of polypharmacy and PIM by care setting. Among home-help users in IC, the prevalence of polypharmacy and PIM was higher (66.8% and 13.0%, respectively) than in SC (60.4% and 11.5%, respectively).

Among those living independently, there was an educational gradient in odds of polypharmacy (Table II). Those with primary (OR=1.31; CI 1.27–1.34) and secondary (OR=1.18; CI 1.15–1.21) education had higher odds of polypharmacy compared to those with post-secondary education. This gradient was not as steep among home-help users or residents in institutions. Females had higher odds of polypharmacy across care need groups. The odds of polypharmacy increased with age among those living independently. However, this pattern was not visible among home-help users or residents in institutions. An increasing CCI score was associated with higher odds of polypharmacy in all care need groups. Those living independently with a dementia diagnosis had higher odds (OR=1.26; CI 1.17–1.36) of polypharmacy, while residents in institutions with dementia had lower odds (OR=0.78; CI 0.71–0.87) compared to those without a dementia diagnosis. Further, those in IC living independently or with home help had higher odds of polypharmacy.

**Table II.** Logistic regression analysis modelling the association between socio-demographic factors and polypharmacy by care need among 65+ years in Stockholm Country.

	Independent				Home-help users				Institutional care			
	Model 0		Model 1		Model 0		Model 1		Model 0		Model 1	
	OR	CI95%	OR	CI95%	OR	CI95%	OR	CI95%	OR	CI95%	OR	CI95%
Education level												
Post secondary	1.00		1.00		1.00		1.00		1.00		1.00	
Primary	1.60	1.56–1.63	1.31	1.27–1.34	1.16	1.05–1.30	1.14	1.02–1.28	1.17	1.05–1.30	1.16	1.03–1.30
Secondary	1.28	1.26–1.31	1.18	1.15–1.21	1.14	1.02–1.26	1.10	0.98–1.23	1.07	0.96–1.17	1.05	0.94–1.18
Sex												
Male	1.00		1.00		1.00		1.00		1.00		1.00	
Female	1.01	0.98–1.02	1.07	1.05–1.09	0.99	0.91–1.08	1.16	1.06–1.28	0.92	0.85–1.01	1.10	1.01–1.21
Age group												
65–74 years	1.00		1.00		1.00		1.00		1.00		1.00	
75–84 years	1.86	1.83–1.90	1.48	1.44–1.51	1.16	1.03–1.37	1.15	1.02–1.31	1.19	1.05–1.36	1.21	1.05–1.39
85+ years	2.49	2.42–2.57	1.59	1.54–1.65	1.05	0.94–1.18	1.06	0.94–1.19	1.05	0.94–1.18	1.05	0.92–1.20
Living situation												
Cohabiting	1.00		1.00		1.00		1.00					
Living alone	1.09	1.07–1.11	0.98	0.96–1.01	0.93	0.84–1.02	0.98	0.88–1.09				
Country of birth												
Sweden	1.00		1.00		1.00		1.00		1.00		1.00	
Other	1.06	1.04–1.09	1.06	1.03–1.09	0.95	0.86–1.06	1.05	0.94–1.17	1.01	0.91–1.12	0.97	0.89–1.09
Care setting												
Standard care	1.00		1.00		1.00		1.00		1.00		1.00	
Integrated care	1.10	1.06–1.15	1.08	1.03–1.09	1.32	1.07–1.62	1.28	1.03–1.59	1.01	0.84–1.20	0.93	0.77–1.12
Morbidity												
CCI score	1.23	1.22–1.24	1.21	1.20–1.22	1.18	1.16–1.21	1.19	1.16–1.22	1.25	1.22–1.29	1.27	1.24–1.31
Dementia diagnosis												
Without	1.00		1.00		1.00		1.00		1.00		1.00	
Dementia	2.31	2.15–2.47	1.26	1.17–1.36	1.05	0.93–1.19	0.93	0.82–1.06	0.96	0.89–1.04	0.78	0.72–0.87

*OR- odds ratio, CI-confidence interval, Reference group-1.00, CCI – Charlson Comorbidity Index *Model 0 = unvariable regression models, *Model 1 = fully adjusted multivariable model (Education, sex, age group, living situation, country of birth, care setting, weighted CCI score, dementia diagnosis). *AIC comparison in Appendix C.

**Table III.** Logistic regression analysis modelling the association between socio-demographic factors and potentially inappropriate medication (PIM) by care need among 65+ years in Stockholm Country.

	Independent				Home-help users				Institutional care			
	Model 0		Model 1		Model 0		Model 1		Model 0		Model 1	
	OR	CI95%	OR	CI95%	OR	CI95%	OR	CI95%	OR	CI95%	OR	CI95%
Education level												
Post secondary	1.00		1.00		1.00		1.00		1.00		1.00	
Primary	1.16	1.11–1.21	1.08	1.03–1.14	0.89	0.75–1.05	0.93	0.78–1.10	1.12	0.96–1.29	1.17	1.00–1.40
Secondary	1.06	1.02–1.10	1.05	1.00–1.10	0.94	0.80–1.11	0.91	0.77–1.08	1.09	0.94–1.26	1.08	0.93–1.27
Sex												
Male	1.00		1.00		1.00		1.00		1.00		1.00	
Female	1.44	1.39–1.49	1.39	1.33–1.44	1.06	0.93–1.21	1.23	1.07–1.42	0.77	0.69–0.86	1.02	0.91–1.15
Age group (years)												
65–74 years	1.00		1.00		1.00		1.00		1.00		1.00	
75–84 years	1.12	1.08–1.16	0.92	0.88–0.96	0.59	0.50–0.68	0.58	0.50–0.69	0.42	0.36–0.48	0.45	0.38–0.52
85+ years	1.03	0.96–1.09	0.70	0.65–0.75	0.37	0.31–0.43	0.36	0.31–0.43	0.26	0.23–0.29	0.28	0.24–0.32
Living situation												
Cohabiting	1.00		1.00		1.00		1.00					
Living alone	1.38	1.33–1.43	1.29	1.24–1.34	0.93	0.81–1.08	0.97	0.83–1.13				
Country of birth												
Sweden	1.00		1.00		1.00		1.00		1.00		1.00	
Other	1.01	0.97–1.06	1.01	0.96–1.06	1.07	0.92–1.25	0.96	0.81–1.13	1.15	1.01–1.31	1.01	0.88–1.17
Care setting												
Standard care	1.00		1.00		1.00		1.00		1.00		1.00	
Integrated care	1.02	0.94–1.10	1.03	0.94–1.12	1.15	0.86–1.54	1.13	0.83–1.53	0.95	0.75–1.20	0.98	0.76–1.25
Morbidity												
CCI score	1.05	1.02–1.06	1.06	1.05–1.07	1.03	1.00–1.06	1.03	1.00–1.06	1.04	1.00–1.05	1.03	0.99–1.06
Dementia diagnosis												
Without	1.00		1.00		1.00		1.00		1.00		1.00	
Dementia	1.18	1.02–1.36	0.98	0.86–1.16	0.50	0.39–0.63	0.53	0.42–0.67	0.50	0.45–0.57	0.52	0.45–0.59

*Abbreviation: OR- odds ratio, CI-confidence interval, Reference group-1.00, CCI – Charlson Comorbidity Index. *Model 0 = unvariable regression models, *Model 1 = multivariable regression model adjusted (Education, sex, age group, living situation, country of birth, care setting, weighted CCI score, dementia diagnosis), *AIC comparison in Appendix C.

Those with primary education had higher odds of PIM (OR=1.08; CI 1.03–1.14; OR=1.17; CI 1.00–1.40) among elderly living independently and in institutional care compared to those with post-secondary education, while education level was not associated with PIM among home-help users. Among those living independently and home-help users, females had higher odds of PIM. Increasing age was associated with lower odds of PIM for all care need groups. Living alone was associated with higher odds of PIM among those living independently but not among home-help users. An increasing CCI score was associated with higher odds of PIM among the independent (OR=1.06; CI 1.05–1.07) but not among residents in institutions and less so among home-help users. There were no significant difference in odds of PIM between the care settings.

Table IV shows the difference in the odds of polypharmacy and PIM stratified by care settings and adjusting for the care need groups. The socio-demographic variables associated with higher odds of polypharmacy were similar in both care settings. However, those in SC born outside of Sweden had higher odds of polypharmacy. In both care settings, female sex, age 85+, living alone and a higher CCI score were associated with higher odds of PIM. There was no significant differences by education level in PIM in IC. However, a social gradient was observed among those in SC. Further, those with greater care needs (with home help and residents in institutions) had higher odds of polypharmacy and PIM compared to those living independently in both care settings.

**Table IV.** Logistic regression analysis modelling the association between socio-demographic factors on polypharmacy and PIM by care setting.

	Polypharmacy				Potentially Inappropriate Medication			
	Standard care		Integrated care		Standard care		Integrated care	
	OR	CI95%	OR	CI95%	OR	CI95%	OR	CI95%
Education level								
Post-secondary	1.00		1.00		1.00		1.00	
Primary	1.30	1.26–1.33	1.19	1.05–1.36	1.09	1.04–1.15	0.94	0.74–1.20
Secondary	1.17	1.14–1.20	1.26	1.11–1.43	1.04	1.00–1.09	1.03	0.83–1.30
Sex								
Male	1.00		1.00		1.00		1.00	
Female	1.07	1.05–1.09	1.13	1.03–1.23	1.31	1.26–1.36	1.41	1.20–1.67
Age groups (years)								
65–74 yrs	1.00		1.00		1.00		1.00	
75–84 yrs	1.47	1.44–1.51	1.49	1.35–1.64	0.88	0.84–0.92	0.91	0.76–1.09
85+ yrs	1.50	1.45–1.55	1.61	1.40–1.85	0.56	0.53–0.60	0.62	0.48–0.81
Living situation								
Cohabiting	1.00		1.00		1.00		1.00	
Alone	0.99	0.96–1.01	1.03	0.93–1.12	1.27	1.23–1.32	1.28	1.08–1.52
Country of birth								
Sweden	1.00		1.00		1.00		1.00	
Other	1.05	1.03–1.08	1.04	0.90–1.09	1.02	0.97–1.07	0.81	0.62–1.07
Morbidity								
CCI score	1.21	1.20–1.22	1.26	1.23–1.29	1.06	1.05–1.07	1.12	1.08–1.16
Dementia diagnosis								
Without	1.00		1.00		1.00		1.00	
Dementia	1.02	0.96–1.07	1.08	0.84–1.38	0.63	0.57–0.69	0.57	0.37–0.89
Care need groups								
Living independently	1.00		1.00		1.00		1.00	
Home help (personal care)	2.16	2.06–2.26	2.42	1.93–2.04	2.03	1.89–2.18	2.02	1.45–2.81
Institutional care	4.48	4.27–4.70	3.60	2.92–4.45	2.39	2.23–2.56	2.19	1.61–2.97

*OR- odds ratio, CI-confidence interval, Reference group-1.00, CCI – Charlson Comorbidity Index *Stratified by care setting Standard vs Integrated care, adjusting for (Education, sex, age group, living situation, country of birth, weighted CCI score, dementia, and care need group.

## Discussion

The emphasis on ‘ageing in place’ and providing community-based care has changed the profile of community-dwelling older people. Previous studies investigating inappropriate prescription drug use have used a binary definition of care need as community dwelling or institutional care [29–33,41–43]. However, this does not reflect the heterogeneity and changing composition of older people in the community. We found that home-help users were more similar demographically in terms of morbidity and prescription drug use to those in institutional care. The prevalence of polypharmacy and PIM was greater among home-help users and residents in institutions. However, socio-demographic differences were more apparent among those living independently, as those who were independent with lower education, older age, female sex, place of birth outside of Sweden, morbidity and dementia had increased odds of polypharmacy and of PIM. Among home-help users and residents in institutions, lower education and morbidity were significant predictors of polypharmacy, while home-help users with a dementia diagnosis had reduced odds of polypharmacy and PIM.

Previous studies observed a higher incidence rate of polypharmacy among those aged 65–74 years and found that polypharmacy persisted in advanced ages [15]. This is comparable to what we observed among those living independently. Further, studies set in institutional care reported that polypharmacy was higher among those aged 75–84 years and decreased among those aged ⩾85 years [14,29]. We found that higher age was associated with lower odds of PIM across care settings. This suggests that the NBHW guidelines for prescribing to people aged ⩾75 years are followed, regardless of care setting [40]. Previous studies have shown that females are prescribed more drugs than males are and that they experience more polypharmacy and PIM [24–26,41]. We observed similar sex differences among those who were independent and home-help users, possibly due to differences in need [22,23]. However, there were fewer sex differences among those in institutions.

We found that lower education was associated with higher odds of polypharmacy, particularly among the independent, consistent with previous studies [25,26]. Educational differences were not as strong in relation to PIM, and they were non-existent for home-help users. The prescribing of PIM is a quality indicator of prescription drug use. Our findings suggest that home help and institutional care might reduce socio-demographic differences, which might be attributed to municipal care being provided based on assessed need rather than being means tested [27]. Further, if older people are experiencing DRP, it might be noticed sooner in this group compared to among those living independently [42].

Consistent with previous studies [6,14,29–31], we found that individuals with a dementia diagnosis in institutions or receiving home help had lower odds of polypharmacy and PIM, whereas individuals with dementia living independently had higher odds of polypharmacy. In Sweden, a dementia diagnosis is a determinant of being admitted into an institution [31]. In this study, a third of institution residents had dementia, being recognised as a vulnerable group requiring 24-hour care. We observed lower odds of polypharmacy and PIM among older people with dementia in institutions than had previously been observed [6]. Further, we also observed this among home-help users, perhaps owing to them being better monitored compared to those living independently. However, other studies have observed lower-quality drug treatment among nursing home residents [29,31]. Moreover, based on the data we have, we cannot determine whether these results are due to under- or over-prescribing in this group [43,44].

Older people exposed to IC should experience improved continuity of care that is more patient oriented and improved communication and information sharing between care professionals. A Canadian study investigated the effect of ‘Care by Design’ (CBD), a coordinated model of primary care, on the rate of polypharmacy and PIM among people in an institution, and found that CBD was associated with a small decrease in the rate of polypharmacy [32]. In contrast, our study found small differences in polypharmacy and PIM between care settings. This might be due to the demographic differences between care settings or the close adherence to national guidelines and priorities regarding appropriate drug prescribing to older people in Sweden, regardless of care setting [40].

### Strengths and limitations

The strengths of this study include that it is population based with a novel approach of comparing the prevalence of different quality indicators of inappropriate pharmaceutical drug use among older people in institutions to community dwellers, distinguishing those with and without home help (i.e. personal care), adjusting for morbidity and dementia diagnoses.

One limitation of this study is the definition of polypharmacy as five or more drugs. Although this is a widely accepted definition, and previous studies have observed that it is often a chronic state, it does not capture those older people who are exposed to the potential harms of polypharmacy sporadically [10]. Further, we were unable to measure older people adherence to complex prescription drug regimes [43], and we could not distinguish between appropriate and inappropriate polypharmacy [44]. Another limitation is that the SPDR, while containing all drugs prescribed and purchased, does not include drugs prescribed and not purchased, or information regarding drugs purchased over the counter or drugs administered in inpatient care [41].

Our definition of PIM is predominantly used in Sweden as an indicator for quality of drug treatment among the elderly [40], which might limit the generalisability of our findings to other countries, as this definition of PIM is stricter than and includes fewer drugs and classes of drugs compared to other criteria, including the PRISCUS list [45], Beers’ criteria [46] and the STOPP-START criteria [47].

## Conclusions

Polypharmacy was associated with care needs and was most prevalent among home-help users and institutional residents, but the socio-demographic differences were most prominent for individuals living independently without home help. This suggests that receiving municipal care may reduce socio-demographic differences. Smaller differences by care needs were found for exposure to potentially inappropriate medication. The care setting (IC or SC) had little effect on the odds for inappropriate drug use, which suggests that national guidelines are followed to the same degree in both settings. Future guidelines should take into consideration how care need can impact inappropriate drug use, particularly for the growing number of older people living independently in the community.

## Supplemental Material

sj-docx-1-sjp-10.1177_14034948211018384 – Supplemental material for Socio-demographic differences in polypharmacy and potentially inappropriate drug use among older people with different care needs and in care settings in Stockholm, Sweden Click here for additional data file. Supplemental material, sj-docx-1-sjp-10.1177_14034948211018384 for Socio-demographic differences in polypharmacy and potentially inappropriate drug use among older people with different care needs and in care settings in Stockholm, Sweden by Megan Doheny, Pär Schön, Nicola Orsini, Johan Fastbom, Bo Burström and Janne Agerholm in Scandinavian Journal of Public Health
