# Supplementary material for: Socio-demographic differences in polypharmacy and potentially inappropriate drug use among older people with different care needs and in care settings in Stockholm, Sweden
Source: Scand J Public Health. 2021 Jun 30;51(1):11–20. doi: 10.1177/14034948211018384 (PMC9903244; doi:10.1177/14034948211018384)
Supplement: sj-docx-1-sjp-10.1177_14034948211018384 – Supplemental material for Socio-demographic differences in polypharmacy and potentially inappropriate drug use among older people with different care needs and in care settings in Stockholm, Sweden [file sj-docx-1-sjp-10.1177_14034948211018384.docx]

**Supplementary Material**

**Appendix A**: Definition of PIM according to the National Board of Health and Welfare’s definition.

|  | **Substance** | **ATC code** |
| --- | --- | --- |
| Long-acting benzodiazepines | Diazepam  Nitrazepam  Flunitrazepam | N05BA01  N05CD02  N05CD03 |
| Tramadol | Tramadol | N02Ax02 |
| Propiomazine | Propiomazine | N05CM06 |
| Drugs with anticholinergic effect | Glycopyrronium bromide  Atropine  Hyoscyamine  Butyl scopolamine  Methyl scopolamine  Scopolamine  Disopyramide  Oxybutynin  Tolterodine  Solifenacin  Darifenacin  Fesoterodine  Morphine and spasmolytic  Ketobemidone and spasmolytic  Trihexyphenidyl  Biperiden  Levmepromazine  Chlorprothixene  Clozapine  Hydroxyzine  Clomipramine  Amitriptyline  Nortriptyline  Maprotiline  Dimenhydrinate  Dexchlorpheniramine  Chlorpheniramine  Alimemazine  Promethazine  Promethazine combinations  Cyproheptadine | A03AB02  A03BA01  A03BA03  A03BB01  A03BB03  A04AD01  C01BA03  G04BD04  G04BD07  G04BD08  G04BD10  G04BD11  NO2AG01  N02AG02  N04AA01  N04AA02  N05AA02  N05AF03  N05AH02  N05BB01  N06AA04  N06AA09  N06AA10  N06AA21  R06AA02  R06AB02  R06AB04  R06AD01  R06AD02  R06AD52  R06Ax02 |

**Appendix B** Defining have a dementia diagnosis

| Alzheimer disease  F00.0 – Alzheimer disease early onset  F00.1 – Alzheimer disease late onset  F00.2 – Alzheimer disease atypical or mixed onset  F00.9 – Alzheimer disease unspecified  G30.0 – Alzheimer disease with early onset  G30.1 – Alzheimer disease with late onset  G30.8 – Other Alzheimer disease  G30.9 – Alzheimer disease unspecified |
| --- |
| Vascular Dementia |
| F01.0 – vascular dementia of acute onset  F01.1 – multi-infarct dementia  F01.2 – sub-cortical vascular dementia  F01.3 – mixed cortical and sub-cortical vascular dementia  F01.8 – other vascular dementia  F01.9 – vascular dementia unspecified |
| Dementia in other disease categories |
| F02.0 – Dementia in Pick disease  F02.1 – Dementia in Jakob disease  F02.2 – Dementia in Huntington disease  F02.3 – Dementia in Parkinson disease  F03.9 – Unspecified Dementia  F03.9Z – Unspecified dementia due to mood disorder  G31.1 – Other degenerative disorders of nervous system  F31.8 – Other recurrent mood disorders |

**Appendix C**

AIC estimates comparing different logistic regression models estimating the association sociodemographic variables and polypharmacy.

|  | Model 1 | Model 2 | Model 3 | Model 4 |
| --- | --- | --- | --- | --- |
|  | AIC | AIC | AIC | AIC |
| Independent | 324260 | 324249.2 | 324245.9 | 211078.8 |
| Home-help | 119017.5 | 118789.3 | 118790.8 | 81727.3 |
| Institution | 16416.2 | 16418.2 | 16420.1 | 14243.3 |
|  |  |  |  |  |
| Model 1 | polypharmacy - education level + age + sex | | | |
| Model 2 | polypharmacy - education level + age + sex + living alone + born outside of Sweden | | | |
| Model 3 | polypharmacy - education level + age + sex + livinag alone+ born outside of Sweden + care setting | | | |
| Model 4 | polypharmacy - education level + age + sex + living alone + born outside of Sweden + care setting + CCI score + dementia diagnosis | | | |
| *For those in institutional care the variable "living alone" was not included  *Model 4, corresponds to Model 1 presented in table 2 | | | | |

AIC estimates comparing different logistic regression models estimating the association sociodemographic variables and being prescribed a potentially inappropriate medication (PIM).

|  | Model 1 | Model 2 | Model 3 | Model 4 |
| --- | --- | --- | --- | --- |
|  | AIC | AIC | AIC | AIC |
| Independent | 13569.7 | 13570 | 13565.6 | 12507.4 |
| Home-help | 7098.7 | 7102.4 | 7103.5 | 6742.4 |
| Institution | 10188.5 | 10190.1 | 10191.6 | 9157.4 |
|  |  |  |  |  |
| Model 1 | PIM - education level + age + sex | | | |
| Model 2 | PIM- education level + age + sex + living alone + born outside of Sweden | | | |
| Model 3 | PIM - education level + age + sex + living alone+ born outside of Sweden + care setting | | | |
| Model 4 | PIM - education level + age + sex + living alone + born outside of Sweden + care setting + CCI score + dementia diagnosis | | | |
| *For those in institutional care the variable "living alone" was not included  *Model 4, corresponds to Model 1 presented in table 3 | | | | |

**Appendix D –** Additional references:

1. Olsson J, Bergman A, Carlsten A, OkÈ T, Bernsten C, Schmidt IK, et al. Quality of drug prescribing in elderly people in nursing homes and special care units for dementia: a cross-sectional computerized pharmacy register analysis. Clin Drug Investig. 2010;30(5):289-300.
2. Andrew MK, Purcell CA, Marshall EG, Varatharasan N, Clarke B, Bowles SK. Polypharmacy and use of potentially inappropriate medications in long-term care facilities: does coordinated primary care make a difference? Int J Pharm Pract. 2018;26(4):318-24.
3. Fog AF, Straand J, Engedal K, Blix HS. Drug use differs by care level. A cross-sectional comparison between older people living at home or in a nursing home in Oslo, Norway. BMC Geriatr. 2019;19(1):49.
4. Goodwin N. How should integrated care address the challenge of people with complex health and social care needs? Emerging lessons from international case studies. Int J Integr Care. 2015;15:e037.
5. Mair et al. Polypharmacy management by 2030: a patient safety challenge. SIMPATHY (Stimulating Innovation Management of Polypharmacy and Adherence in The Elderly) 2017.
6. Kodner DL, Spreeuwenberg C. Integrated care: meaning, logic, applications, and implications – a discussion paper. Int J Integr Care 2002;2:1–6.
7. Doheny M, Agerholm J, Orsini N, Schön P, Burström B. Impact of integrated care on trends in the rate of emergency department visits among older persons in Stockholm County: an interrupted time series analysis. BMJ Open. 2020;10(6):e036182.
8. Bäck MA, Calltorp J. The Norrtälje model: a unique model for integrated health and social care in Sweden. Int J Integr Care. 2015;15:e016.
9. Ludvigsson JF, Appelros P, Askling J, Byberg L, Carrero JJ, Ekström AM, et al. Adaptation of the Charlson Comorbidity Index for Register-Based Research in Sweden. Clin Epidemiol. 2021;13:21-41.
10. Swedish National Board of Health and Welfare. Indikatorer för god läkemedelsterapi hos äldre, 2010. Available: https://www.socialstyrelsen. se/ publikationer2010/ 2010- 6- 29 [Accessed Nov2020)
11. Ringbäck Weitoft G E, FJ. Prescription Drugs, Health in Sweden: The National Puvlix Health Report 2012. Chapter 18. Scandanavian Journal of Public Health. 2012;40:294-304.
12. Zelko E, Klemenc-Ketis Z, Tusek-Bunc K. Medication Adherence in elderly with polypharmacy living at home: A systematic review of existing studies. Mater Sociomed. 2016;28(2):129-32.
13. Kuijpers MA, van Marum RJ, Egberts AC, Jansen PA, Group OOpDdS. Relationship between polypharmacy and underprescribing. Br J Clin Pharmacol. 2008;65(1):130-3.
14. Cadogan CA, Ryan C, Hughes CM. Appropriate Polypharmacy and Medicine Safety: When Many is not Too Many. Drug Saf. 2016;39(2):109-16.
15. Holt S, Schmiedl S, Th¸rmann PA. Potentially inappropriate medications in the elderly: the PRISCUS list. Dtsch Arztebl Int. 2010;107(31-32):543-51.
16. Panel BtAGSBCUE. American Geriatrics Society 2019 Updated AGS Beers CriteriaÆ for Potentially Inappropriate Medication Use in Older Adults. J Am Geriatr Soc. 2019;67(4):674-94.
17. O'Mahony D, O'Sullivan D, Byrne S, O'Connor MN, Ryan C, Gallagher P. STOPP/START criteria for potentially inappropriate prescribing in older people: version 2. Age Ageing. 2015;44(2):213-8.
